# Genetic connectivity and population expansion inferred from multilocus analysis in *Lutjanus alexandrei* (Lutjanidae–Perciformes), an endemic snapper from Northeastern Brazilian coast

**DOI:** 10.7717/peerj.15973

**Published:** 2023-09-26

**Authors:** Ivana Veneza, Raimundo da Silva, Charles Ferreira, Patrícia Mendonça, Iracilda Sampaio, Grazielle Evangelista-Gomes

**Affiliations:** 1 Campus Universitário de Monte Alegre, Universidade Federal do Oeste do Pará, Monte Alegre, Pará, Brazil; 2 Instituto de Estudos Costeiros/Laboratório de Genética Aplicada, Universidade Federal do Pará, Bragança, Pará, Brazil; 3 Instituto de Estudos Costeiros/Grupo de Genética e Conservação, Universidade Federal do Pará, Bragança, Pará, Brazil; 4 Instituto de Estudos Costeiros/Laboratório de Genética e Biologia Molecular, Universidade Federal do Pará, Bragança, Pará, Brazil

**Keywords:** Genetic diversity, Endemicity, Atlantic Ocean, Population structure, Snapper

## Abstract

Previous studies about the genetic diversity, connectivity and demographic history in Lutjanidae fishes have reported a common pattern of genetic homogeneity and expansion in populations from Western South Atlantic. In the present work, we inferred the population structure, the levels of genetic diversity and the demographic history of the Brazilian snapper *Lutjanus alexandrei*, a recently described and endemic species from Northeastern coast of Brazil. Five different fragments, including mitochondrial DNA (Control Region, Cyt b and ND4) and nuclear DNA (Myostatin and S7) regions were analyzed in 120 specimens of *L. alexandrei* from four localities in Northeastern Brazil, representing the first study of population genetics in this species. High levels of genetic diversity were observed following a panmictic pattern, probably related to the larval dispersal by the current tides along the Brazilian coast. In addition, both demographic history and neutrality tests indicated that *L. alexandrei* has undergone population expansion during Pleistocene. In this sense, the sea level variation from this period could have increased the available resources and suitable habitats for the Brazilian snapper.

## Introduction

Several studies focusing on the population structure, demographic history and genetic diversity have been carried in Lutjanidae species (snappers) from Western Atlantic. While population substructure has been commonly reported in several species of snappers from Northern Atlantic regions, such as the Caribbean and Gulf of Mexico (Vasconcellos et al., 2008; Karlsson, Saillant & Gold, 2009; Carson et al., 2011; Saillant et al., 2012; Silva et al., 2015; Silva et al., 2018), high levels of genetic variation, population expansion and panmixia have been detected in snappers from the Brazilian coast (Silva et al., 2015; da Silva et al., 2016; Souza et al., 2019; Evangelista-Gomes et al., 2020). The only exception for this pattern so far was identified in *Lutjanus synagris*, characterized by low levels of genetic polymorphism along Western South Atlantic (Silva et al., 2018; Evangelista-Gomes et al., 2020).

Life traits, such as larval dispersal abilities, might play a major role in the lack of population structure, resulting in genetically similar populations across their range (Lindeman et al., 2001), as suggested for snappers from the Brazilian coast, like *L. synagris* (Silva et al., 2018), *L. purpureus* (da Silva et al., 2016), *O. chrysurus* (Vasconcellos et al., 2008; Silva et al., 2015), *L. analis* and *L. jocu* (Souza et al., 2019).

*Lutjanus alexandrei*, popularly known as Brazilian snapper, Baúna or Baúna-de-fogo is a recently described species of Lutjanidae from South Atlantic whose distribution is more restricted than other snappers, being endemic to Northeastern Brazil, from the coast of Maranhão to Bahia (Moura & Lindeman, 2007). Moreover, this species is commonly found in estuaries, particularly at juvenile stages, suggesting that the Brazilian snapper is an estuarine-dependent fish (Aschenbrenner & Ferreira, 2015; Aschenbrenner, Hackradt & Ferreira, 2016). Nonetheless, many life traits of this species remain poorly known.

Taking into account the snappers described along the Brazilian coast, *L. jocu* (distributed from Northern to Southeastern Brazil) is closely related to the Brazilian snapper (Veneza et al., 2019), being both species observed in sympatry along the Northeastern coast. *L. jocu* is also considered an estuarine-dependent species, including mangrove areas (Moura & Lindeman, 2007; Osório, Godinho & Lotufo, 2011; Aschenbrenner, Hackradt & Ferreira, 2016). A previous genetic study in *L. jocu* from the Brazilian coast based on mitochondrial and nuclear DNA regions revealed a similar pattern reported in other snappers from this region, *i.e*., high levels of diversity and gene flow, and population expansion starting from the Pleistocene (Souza et al., 2019).

Because of the relatively restricted range when compared to congeners, a strictly estuarine-dependent juvenile stage and the lack of information about their evolutionary history, detailed studies for populations of *L. alexandrei* are required to infer their conservation status. This information is essential to design effective management plans taking into account the endemicity of this species and the history of fisheries exploitation in snappers from the Brazilian coast (IBAMA, 2000–2007; Ministry of Fishing and Aquaculture of Brazil (MPA), 2008–2010), including overexploitation of local stocks in Northeastern Brazil (Frédou, Ferreira & Letourneur, 2009).

Therefore, the goal of this study was to provide a panorama of the population structure, demographic history and genetic diversity of *L. alexandrei* that along with the available information about the life traits might provide insights for effective management of the Brazilian snapper as well as inferences about the phylogeographic patterns and processes that influenced the distribution of genetic variation and connectivity of this species along the Brazilian coast.

## Materials and Methods

### Sampling

Samples from 120 specimens of *L. alexandrei* were obtained between 2013 and 2018. Some of these samples were kindly provided by colleagues from muscle tissues stored at their facilities. The remaining samples were obtained from dead individuals, procured through direct purchase from specimens locally caught and commercially sold by artisanal fishermen. Therefore, there was no need to apply for a license for collection or approval by the Animal Ethics Committee. All samples were collected/transported with authorization of the Instituto Chico Mendes de Conservação da Biodiversidade (ICMBio) (SISBIO license n. 62250-1).

**Figure 1:**
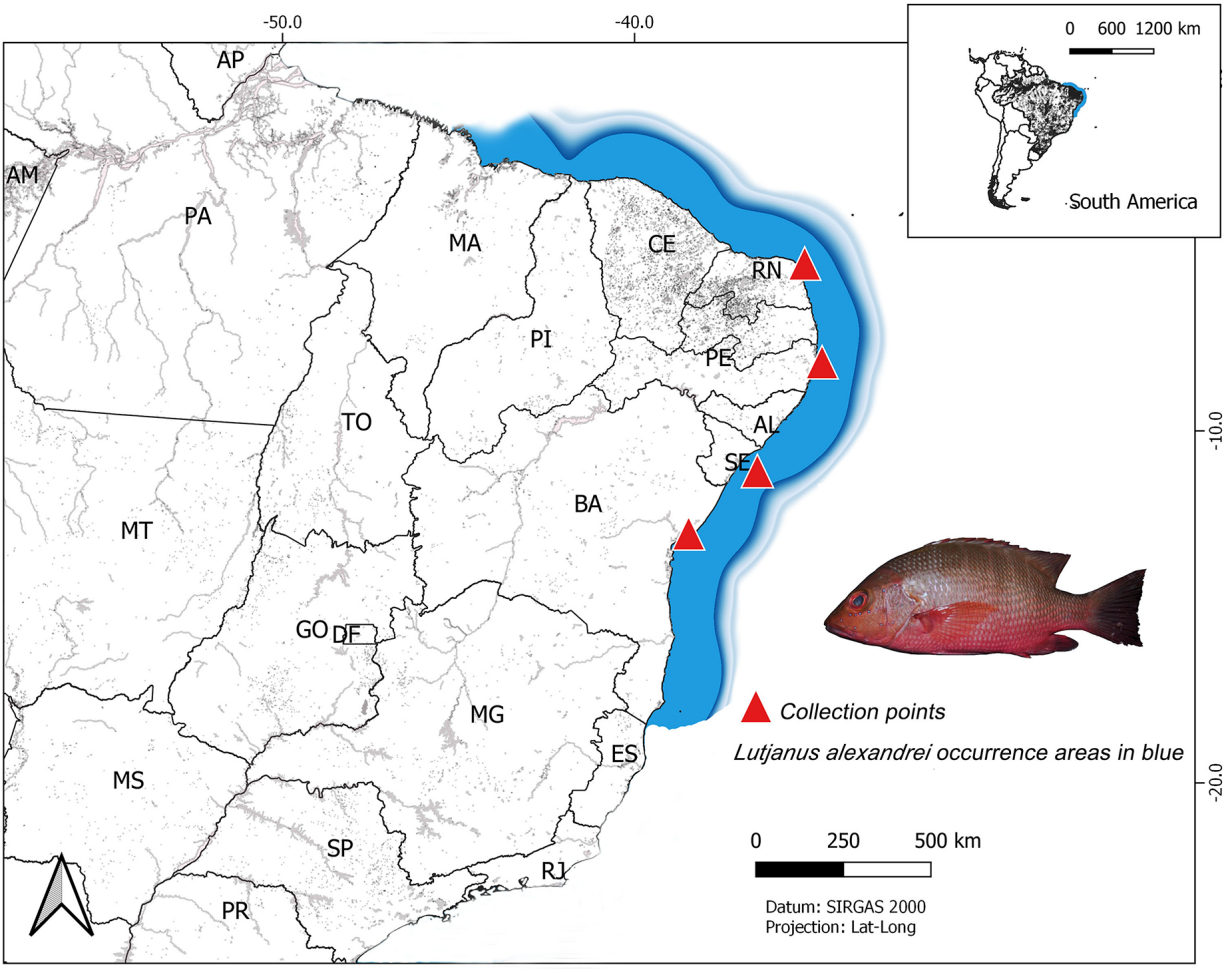
Map of South America showing the collect sites on the Brazilian northeast coast of the specimens of *Lutjanus alexandrei* analyzed in the present study. The area shaded in blue represents the natural range of the Brazilian snapper.

The analyzed specimens were obtained from four localities in Northeastern Brazilian coast: Salvador (Bahia–BA) (*n* = 14; all individuals were juveniles and were captured near beaches and provided by artisanal fishermen, in 2018); Aracaju (Sergipe–SE) (*n* = 28; all individuals were juveniles and sampled in 2018, most were obtained from fish markets; three specimens were captured by artisanal fishermen in the Vaza Barris river estuary); Tamandaré (Pernambuco–PE) (*n* = 31; all samples were provided from the analysis material of Rodrigo Bastos, a postdoctoral student supervised by professor Beatrice Padovani. The fish were captured in 2017, in the estuary of the Ariquindá River, on the coastal reefs, in Vau de Tamandaré and Sirinhaém beach) and Nísia Floresta (Rio Grande do Norte–RN) (*n* = 48; most individuals were juveniles and were collected in the estuary of the Pium River or in coastal reefs of the Praia de Pirangi, by artisanal fishermen, in 2013, 2014 and 2018. Some adult specimens were sampled in markets on Ponta Negra Beach in 2014) (Fig. 1). Their taxonomic identification was carried out using the specialized literature (Moura & Lindeman, 2007) and the samples of muscle tissue were tagged and stored in microtube with 100% ethanol at −20 °C up to laboratory procedures. Voucher specimens were deposited in the ichthyological collection from the Laboratory of Applied Genetics at Universidade Federal do Pará, *Campus* of Bragança, Pará.

### Laboratory procedures

Total DNA was isolated using the commercial Wizard Genomic® kit (Promega, Madison, WI, USA), according to the manufacturer’s instructions. The quality of the extract DNA was verified by electrophoresis in 1% agarose gel stained with Gel Red™ (Biotium, Fremont, CA, USA) and exposed to UV light. The amplification of the fragments of interest was performed *via* PCR (polymerase chain reaction). The analyzed sequences comprised mitochondrial DNA (mtDNA) regions—Cytochrome b (Cyt b), NADH dehydrogenase subunit 4 (ND4) and Control Region (CR)—and nuclear DNA genes (intron 1 of the Myostatin and intron 1 of the S7 ribosomal protein).

The PCRs comprised 2.4 μL of dNTPs (1.25 mM), 1.5 μL of buffer (10x), 0.6 μL of MgCl_2_ (50 mM), 0.6 μL of each primer (50 ng/μL), 0.1 μL of Taq DNA polymerase (U/μL), about 50 ng of template DNA and ultrapure water to a final volume of 15 μL. The primers used to amplify each fragment of interest are described in Table 1.

**Table 1:** Primers used to amplify mitochondrial and nuclear DNA fragments along with their respective references and annealing temperatures.

Marker	Primers-sequence 5′-3′	Reference	Annealing (°C)
Cyt B	FishCybF-ACCACCGTTGTTATTCAACTACAAGAAC	Sevilla et al. (2007)	54
	TrucCytbR-CCGACTTCCGGATTACAAGACCG		
ND-4	NAP2-CAAAACCTTAATCTYCTACAATGCT	Arevalo, Davis & Sites (1994)	56
	ND4LB-CAAAACCTTAATCTYCTACAATGCT	Bielawski & Gold (2002)	
CR	A-TTCCACCTCTAACTCCCAAAGCTAG	Lee et al. (1995)	57
	G-CGTCGGATCCCATCTTCAGTGTTATGCTT		
Myostatin	Myo 1F-ATGAGCATGCCATCACAGAG	Silva et al. (2017)	60
	Myo 1R-ATGCGATTGGCTTGAAACTT		
S7	S7RPEX1F-TGGCCTCTTCCTTGGCCGTC	Chow & Hazama (1998)	57
	S7RPEX2R-AACTCGTCTGGCTTTTCGCC		

The PCR conditions encompassed a first denaturation step at 94 °C for 3 min; followed by 35 cycles of denaturation at 94 °C for 40 s, annealing at specific temperature for each primer for 40 s (Table 1), and extension at 72 °C for 2 min plus a final extension step at 72 °C for 2 min.

The amplicons were purified in PEG 8000 (polyethylene glycol) according to Paithankar & Prasad (1991) and sequenced by the dideoxy method (Sanger, Nicklen & Coulson, 1977) using the Big Dye kit (ABI Prism™ Dye Terminator Cycle Sequencing Reading Reaction; Thermo Fisher, Waltham, MA, USA). The final products were read in ABI 3500 (Thermo Fisher, Waltham, MA, USA) automatic sequencer.

### Dataset

The sequences were organized into individual datasets for each marker and visualized, edited and corrected in the software BioEdit (Hall, 1999). Each sequence dataset was aligned using CLUSTAL W (Thompson, Higgins & Gibson, 1994), available in Bioedit.

Heterozygous individuals were detected when double peaks were identified on both strands in the electropherograms. The gametic phase was determined using Phase v. 2.1 (Stephens, Smith & Donnelly, 2001), available in DNAsp v. 5.10 (Librado & Rozas, 2009). Three runs with distinct random seeds, 1,000 iterations per chain, thinning interval equal to 1, and a burn-in of 1,000 were carried out. Only the haplotypes with probability values above 0.59 were selected for the further analyses.

The occurrence of intragene recombination was estimated by the PhiW test (Bruen, Philippe & Bryant, 2006) using the software Splits Tree v. 4.6 (Huson & Bryant, 2006). Whenever present, a dataset free of recombination was obtained using the software Imgc (Woerner, Cox & Hammer, 2007).

### Population structure

The genetic polymorphism in populations of *L. alexandrei* was estimated based on the values of haplotype (h) and nucleotide (π) diversity (Nei, 1987) using the software Arlequin v. 3.5 (Excoffier & Lischer, 2010).

To infer about the spatial distribution of haplotypes and their genealogical relationships, haplotype networks were built for each genomic region in the software Haploviewer (Salzburger, Ewing & Von, 2011). For that, we used as input a tree topology inferred from Maximum Likelihood, available in the DNAml package and implemented in Phylip 3.6 (Felsenstein, 2005).

The analysis of molecular variance (AMOVA) (Excoffier & Lischer, 2010) was carried out to test the population subdivision of *L. alexandrei* along their range based on the frequency and number of mutations among haplotypes (Excoffier & Lischer, 2010). The AMOVA was performed considering the samples from distinct localities as a single group. The significance of fixation indexes was estimated using 10,000 non-parametric permutations. The level of genetic differentiation among the sampled localities was estimated from pairwise FST values as calculated by Weir & Hill (2002) and the significance of this result was based on 10,000 bootstrap permutations assuming a significance level of 0.05.

The population structure was also evaluated using a Bayesian clustering analysis in the software Bayesian analysis of genetic population structure (BAPS) v. 6.0 (Corander et al., 2013). This method allows identifying the genetic groups independently on the geographic distribution of samples. In this study, BAPS was performed assuming an admixture model among specimens from all collection sites with linked loci and up to 20 putative clusters (k = 20).

### Neutrality tests and demographic history

The neutrality of the analyzed loci was evaluated using Tajima’s (1989) D and Fu’s (1997) Fs tests in Arlequin v. 3. 5 (Excoffier & Lischer, 2010) based on 10,000 permutations for estimating the *p* significance values.

The Extended Bayesian Skyline Plot (EBSP) (Heled & Drummond, 2008) available in the software Beast v.1.8 (Drummond et al., 2012) was also used to evaluate the population dynamics through time, allowing integrating multilocus data. Each run comprised 500 million MCMC iterations, sampled at every 10,000 iterations, with a burn-in of 10%.

The best evolutionary models were estimated using the software PartitionFinder 2.1 (Lanfear et al., 2016), as follows: HKY+I+G (CR) and TRN+I+G (Cyt b; ND4; Myostatin and S7). We assumed a relaxed clock with an uncorrelated lognormal distribution and found that the markers’ ucld.stdev values were close to zero, indicating that the uniform replacement rate hypothesis was not rejected. Then strict clock and evolutionary rate of 3.6% per million years for CR (Donaldson & Wilson, 1999; da Silva et al., 2020); 1% for Cyt b and ND4 (Bowen et al., 2001; Lessios, 2008; Gaither et al., 2010; Delrieu-Trottin et al., 2017; Da Cunha et al., 2020); 0.4% for S7 intron I (Janko et al., 2007; Delrieu-Trottin et al., 2017); and for Myostatin we estimate the rate. Was employed in the software Beast v.1.8 using the Cipres Science Gateway v. 3.3 platform (Miller, Pfeiffer & Schwartz, 2010) available in http://www.phylo.org/index.php/portal/cite_us. After finishing the run, ESS >200 values were verified in TRACER 1.7.1 (Rambaut et al., 2018) and the graph was generated using the Python script provided by Heled (2010).

## Results

### Characterization of the dataset and genetic diversity

Five different fragments from mtDNA and from nuclear DNA were sequenced in the present study, totaling 2,997 base pairs (bp), being 2,084 bp related to mtDNA and 913 pb from nuclear DNA.

The verification of the occurrence of intragenic recombination showed that, in contrast to the myostatin intron 1, recombination was detected for the fragment of the intron 1 of S-7 ribosomal protein.

As for the mitochondrial fragments, for CR (mtDNA), a bank of sequences with a length of 806 bp was obtained for 111 specimens (accession numbers: MZ327145–MZ327255). From this total, we recovered 110 haplotypes and high levels of genetic diversity across the sampled area which can be observed by the values obtained for haplotype diversity (h = 1.0 for the entire range of samples) and for nucleotide diversity (π ranging from 0.033 in Bahia to π = 0.041 in Rio Grande do Norte). For the CytB, sequences of 808 bp were obtained for 110 specimens (accession numbers: MZ343890–MZ343999), comprising 25 haplotypes and haplotype diversity (h) ranging from 0.1818 in Bahia to h = 0.8376 in Pernambuco while the nucleotide diversity (π) varied from 0.001 and 0.002 for the same localities. The ND4 fragment, woth470 bp for 117 individuals (accession numbers: MZ356036–MZ356152), encompassed 23 haplotypes and h values from 0.6527 for the samples from Pernambuco to 0.9103 in Bahia, locations in which nucleotide diversity for this fragment ranged from π = 0.002 to π = 0.003, respectively (Table 2).

**Table 2:** Summary statistics about the genetic diversity and neutrality tests in populations of *Lutjanus alexandrei*.

*Cytochrome b–808 pb*							
	N	H	S	h	π	Tajima’s D	Fu’s Fs
Rio Grande do Norte	44	13	12	0.7822	0.001676	−1.54055*	−8.04383*
Pernambuco	27	11	11	0.8376	0.001996	−1.43306	−5.91584*
Sergipe	28	12	12	0.8042	0.001981	−1.59303	−7.31668
Bahia	11	2	2	0.1818	0.000450	−1.42961	0.50645*
Total	110	25	22	0.7593	0.001707	−1.94125*	−23.67698*

*Control region–806 pb*							
	N	H	S	h	π	Tajima’s D	Fu’s Fs
Rio Grande do Norte	46	46	189	1.0000	0.040731	−0.88204	−22.48052*
Pernambuco	26	26	154	1.0000	0.040331	−0.81116	−8.14714
Sergipe	28	28	148	1.0000	0.040308	−0.64305	−9.34247*
Bahia	11	11	93	1.0000	0.032636	1.00193	−1.71508
Total	111	110	243	0.9998	0.039269	−1.06290	−23.93175*

*NADH-dehydrogenase subunit 4–470 pb*							
	N	H	S	h	π	Tajima’s D	Fu’s Fs
Rio Grande do Norte	48	12	12	0.7766	0.002652	−1.60572*	−6.86688*
Pernambuco	29	8	6	0.6527	0.001866	−1.22619	−4.64105*
Sergipe	27	8	8	0.7949	0.002691	−1.21654	−3.20917*
Bahia	13	8	7	0.9103	0.003437	−1.07663	−4.49954*
Total	117	23	20	0.7689	0.002544	−1.93438*	−21.81913*

*Intron 1 of the S7 ribosomal protein–506 pb*							
	N	H	S	h	π	Tajima’s D	Fu’s Fs
Rio Grande do Norte	31	28	20	0.9651	0.007227	−0.43636	−17.39503*
Pernambuco	14	14	13	0.9392	0.007670	0.54201	−4.18168
Sergipe	20	23	19	0.9654	0.007011	−0.67670	−15.01621*
Bahia	5	8	9	0.9556	0.005753	−0.37198	−3.65941*
Total	70	36	20	0.9581	0.007133	−0.01307	−20.37711*

*Intron 1 of the Myostatin–407 pb*							
	N	H	S	h	π	Tajima’s D	Fu’s Fs
Rio Grande do Norte	47	5	4	0.5404	0.001764	−0.16365	−0.50836
Pernambuco	30	6	4	0.6113	0.002053	−0.05646	−1.39549
Sergipe	23	7	5	0.5990	0.002410	−0.34041	−2.16023
Bahia	12	5	4	0.7101	0.002582	−0.05216	−0.75801
Total	112	9	7	0.5886	0.002051	−0.59907	−2.81631

**Notes:**

* *p* value.

N, number of specimens; H, number of haplotypes; S, number of polymorphic sites; h, haplotype diversity; π, nucleotide diversity.

With regard to nuclear genome fragments, for intron I S7 a total of 36 haplotypes were recovered from the 506 bp sequenced for 70 specimens (accession numbers: MZ355926–MZ356035), in addition to allelic diversity values above 0.9 for all populations and nucleotide diversity ranging from π = 0.006 in Bahia to π = 0.008 in Pernambuco. At last, the myostatin intron 1 comprised sequences of 407 bp for 112 specimens (accession numbers: MZ344000–MZ344114), distributed into nine haplotypes. The h values for this fragment varied from 0.5404 to 0.7101, while the π values varied from 0.002 to 0.003 for the samples from Rio Grande do Norte and Bahia, respectively (Table 2).

### Population genetic structure

**Figure 2:**
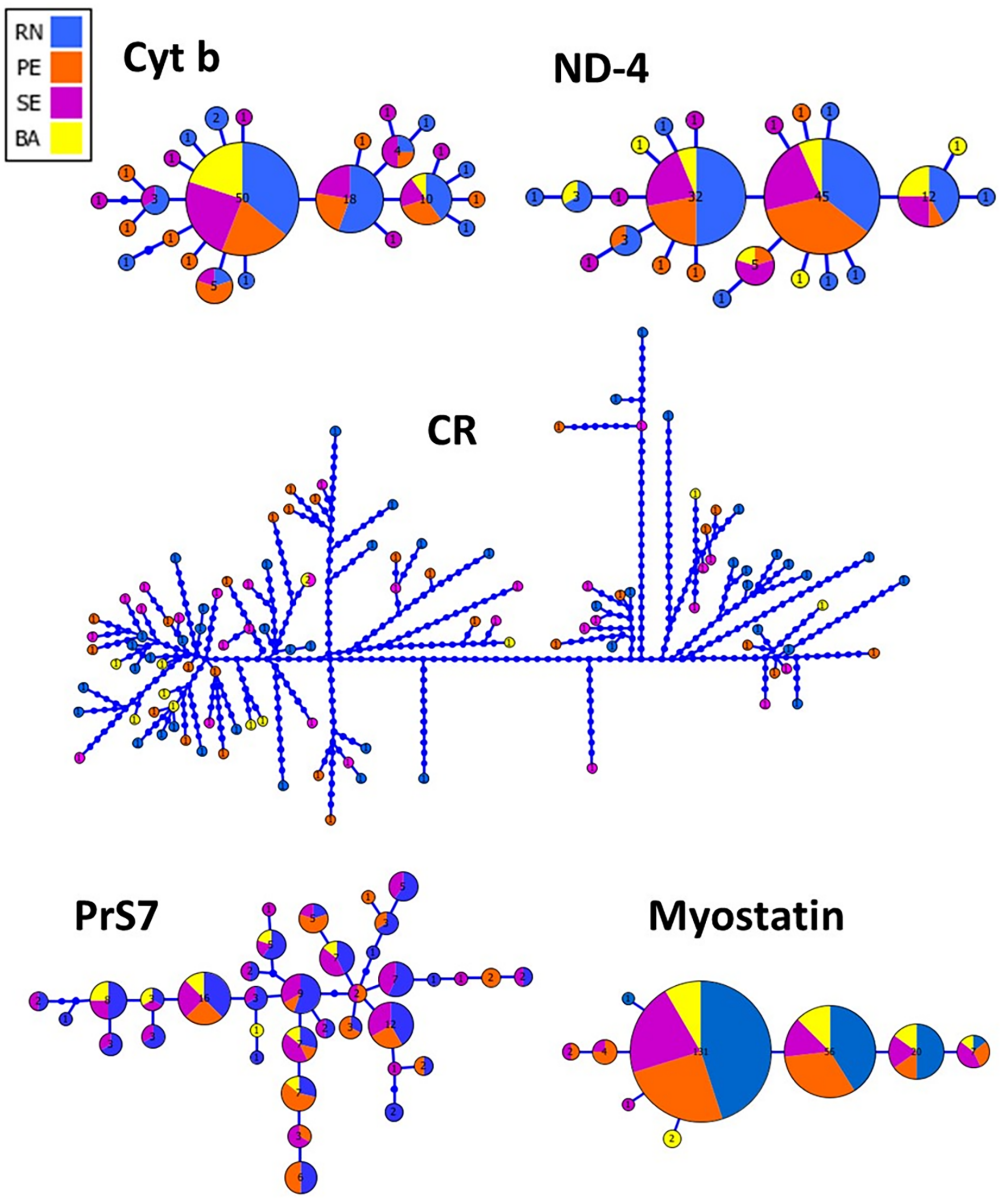
Genealogical relationships among the haplotypes of *Lutjanus alexandrei* for the loci used in the present study and their geographic distribution (Cyt b, Cytochrome b; ND-4, NADH dehydrogenase subunit 4; CR, Control Region; PrS7, Intron 1 of the S7 ribosomal protein; Myostatin, intron 1 of the Myostatin). The size of the circles refers to the frequency of each haplotype.

The haplotype network inferred from both mitochondrial and nuclear markers revealed a remarkable admixture pattern with homogeneous distribution across the sampled area in Northeastern Brazil. For the CR, a single haplotype was shared by two specimens while the others represented unique haplotypes. A total of 25 haplotypes were recovered using Cyt B with the three most frequent haplotypes being shared by 50, 18 and 10 specimens, respectively, while the remaining haplotypes showed low frequency or were unique, similarly to the pattern observed in ND4. Three haplotypes were also the most frequent ones for the Myostatin intron 1, being present in 131, 56 and 20 specimens, respectively, from all populations sampled in this study. The haplotype network for the intron 1 of S7 ribosomal protein revealed a more patched scenario, with two frequent haplotypes shared among entre 16 and 12 specimens, while the others were observed at low frequencies (Fig. 2).

This lack of population subdivision among the sampled populations was confirmed by AMOVA (Table 3), since most of genetic variation was observed within populations, as also reinforced by F/θST values. In addition, these results were supported by BAPS inasmuch as a single cluster (K = 1) presented the maximum probability value.

**Table 3:** Analysis of molecular variance (AMOVA) for populations of *Lutjanus alexandrei* along the Northeastern coast of Brazil.

*Cytochrome b*		
Source of variation	Variation (%)	Φst
Within of populations	−0.80164	0.00000
Among groups	100.80164	

*Control region*		
Source of variation	Variation (%)	Φst
Within of populations	−0.87623	0.00000
Among groups	100.87623	

*NADH-dehydrogenase subunit 4*		
Source of variation	Variation (%)	Φst
Within of populations	−0.30726	0.00000
Among groups	100.30726	

*Intron 1 of the S7 ribosomal protein*		
Source of variation	Variation (%)	Φst
Within of populations	−0.46137	0.00000
Among groups	100.46137	

*Intron 1 of the Myostatin*		
Source of variation	Variation (%)	Φst
Within of populations	−0.55904	0.00000
Among groups	100.55904	

Finally, the pairwise FST values among the localities corroborated the homogeneity among populations of *L. alexandrei*, as revealed by low and non-significant values for all studied fragments (Table 4).

**Table 4:** Pairwise Fst comparison in populations of *Lutjanus alexandrei* from the Northeastern coast of Brazil.

*Cytochrome b*				
Locality	Sergipe	Bahia	Pernambuco	Rio Grande do Norte
Sergipe	–			
Bahia	0.02918	–		
Pernambuco	−0.01890	0.02153	–	
Rio Grande do Norte	−0.01713	0.02863	0.01829	–

*Control region*				
Locality	Sergipe	Bahia	Pernambuco	Rio Grande do Norte
Sergipe	–			
Bahia	0.00005	–		
Pernambuco	−0.02397	0.00773	–	
Rio Grande do Norte	−0.01198	0.01681	−0.00913	–

*NADH-dehydrogenase subunit 4*				
Locality	Sergipe	Bahia	Pernambuco	Rio Grande do Norte
Sergipe	–			
Bahia	0.00416	–		
Pernambuco	−0.02099	0.04667	–	
Rio Grande do Norte	−0.01576	0.01631	−0.00743	–

*Intron 1 of the S7 ribosomal protein*				
Locality	Sergipe	Bahia	Pernambuco	Rio Grande do Norte
Sergipe	–			
Bahia	0.01759	–		
Pernambuco	−0.01273	0.04153	–	
Rio Grande do Norte	−0.01156	0.00643	−0.00605	–

*Intron 1 of the Myostatin*				
Locality	Sergipe	Bahia	Pernambuco	Rio Grande do Norte
Sergipe	–			
Bahia	−0.00961	–		
Pernambuco	−0.00872	−0.00485	–	
Rio Grande do Norte	−0.00768	−0.00087	−0.00362	–

### Neutrality and demographic history

Both neutrality tests (Tajima’s D and Fu’s Fs) showed negative and significant values for most of populations and considering all fragments included in this study. Regarding the mitochondrial DNA fragments included in this study, for the Control Region, Tajima’s D values ranged from −0.88204 in Rio Grande do Norte to 1.00193 in Bahia, while for Fu’s Fs values ranged from −22.48052 in Rio Grande do Norte and −1.71508 for the same locations. For Cytochrome b, the variation was from −1.59303 in Sergipe to −1.42961 in Bahia, and from −8.04383 in Rio Grande do Norte to 0.50645 in Bahia, for Tajima’s D and Fu’s Fs, respectively. The ND4 fragment analyzed reported a variation from −1.60572 in Rio Grande do Norte to −1.07663 in Bahia in Tajima’s D values and in relation to the Fu’s Fs test, the variation was in the range of −6.86688 in Rio Grande do Norte at −3.20917 in Sergipe (Table 2).

As for nuclear fragments, Intron 1 of the S7 ribosomal protein showed Tajima’s D values between −0.67670 in Sergipe and 0.54201 in Pernambuco, while the variation in the Fu’s Fs test was between −17.39503 and −3.65941, for Rio Grande do Norte and Bahia. Finally, Tajima’s D values found for Intron 1 of the Myostatin comprised the range of −0.34041 to −0.05216 for Sergipe and Bahia, respectively; for Fu’s Fs the variation ranged from −2.16023 in Sergipe to −0.50836 in Rio Grande do Norte (Table 2).

**Figure 3:**
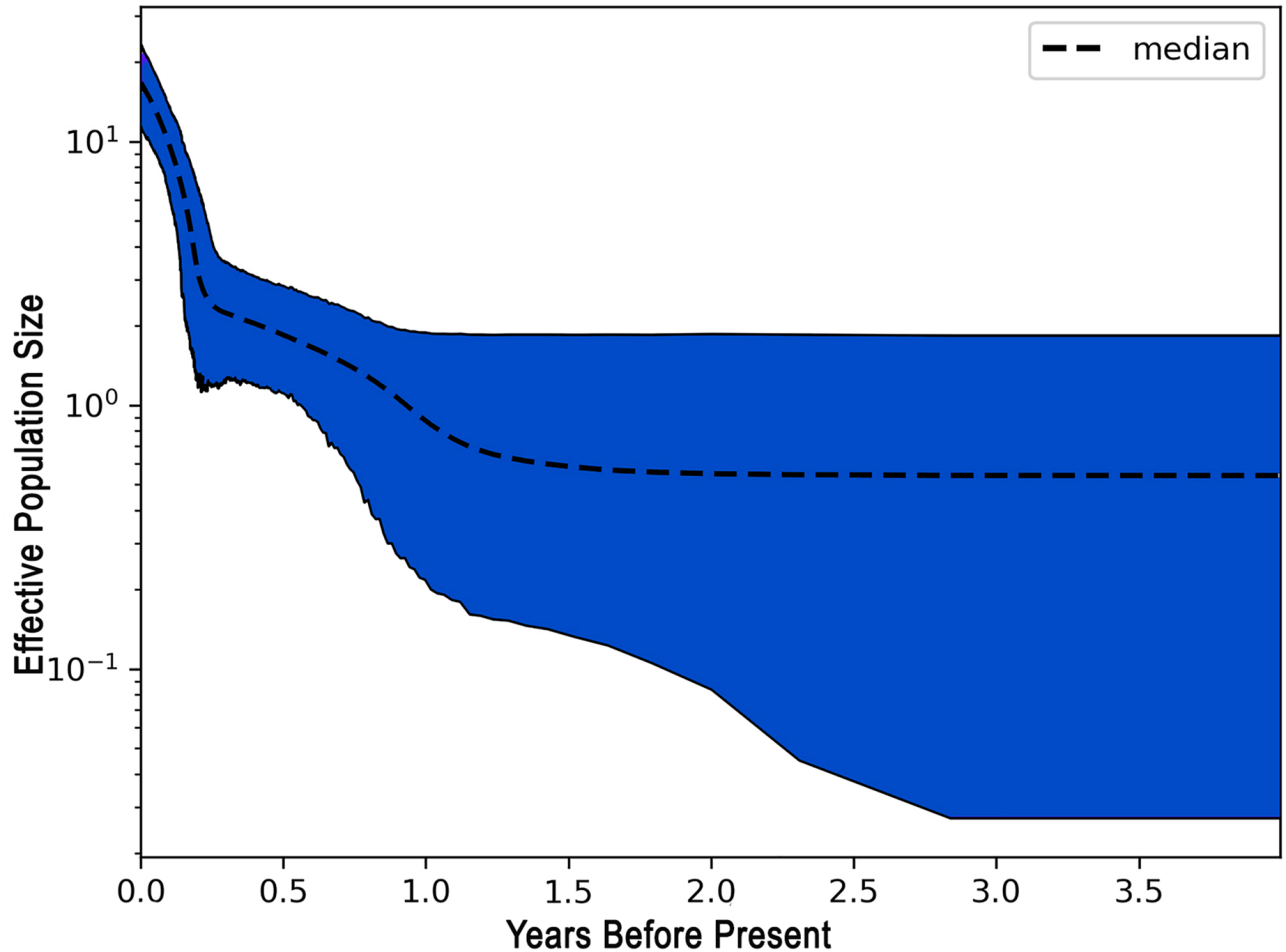
Extended Bayesian skyline plot (EBSP) graph recovered for *Lutjanus alexandrei* based on multiloci analyses in samples from Northeastern Brazil. The horizontal axis indicates the time scale in million years.

The demographic history of *L. alexandrei* throughout the Northeastern coast of Brazil was investigated using the EBSP approach. The multilocus analysis resulted in a skyline plot indicating increases of the effective population size during Pleistocene, around 800 thousand years ago (Fig. 3).

## Discussion

### Genetic diversity

Nuclear introns are expected to be more suitable for phylogenetic analysis than population-genetics, due to the slow rate of evolution, among other issues. However, to obtain a more complete and realistic scenario, the inclusion of polymorphic sequences is necessary, considering that the use of mitochondrial markers and microsatellite sequences, widely used and recognized as useful, have limitations that indicate the need to use other genomics regions to complement their results, leading fragments of nuclear DNA to be applied to studies of population genetics, with introns being the best candidates (Zhang & Hewitt, 2003).

In general, the analyses of the genomic regions in this study revealed high levels of genetic variation in *L. alexandrei*, particularly in relation to CR. This pattern has been commonly reported in snappers from the Brazilian coast, such as *L. purpureus* (h = 0.997; π = 0.029) (da Silva et al., 2016), *O. chrysurus* (h = 0.963; π = 0.0179) (da Silva et al., 2015), *L. analis* (h = 0.9962; π = 0.0315) and *L. jocu* (h = 0.9962; π = 0.,0362) (Souza et al., 2019). Nonetheless, the diversity herein reported in *L. alexandrei* is higher than that observed in the abovementioned species.

In the case of snappers, highly targeted by commercial fisheries, such high values of genetic diversity are unexpected since several overexploited marine species have shown a reduction of genetic variation (Pinsky & Palumbi, 2014). On the other hand, the fisheries pressure on Lutjanidae is a recent process, dating back about 50 years ago (Fonteles-Filho, 1972). Therefore, the negative effects of this activity might remain undetected by genetic analyses even if populations of snappers have been impacted by overexploitation. In addition, some fragments such as the CR are recognized by indicating diversity losses related to past events, such as historic reduction of effective population size, as reported for other marine fishes like *Merluccius paradoxus* (Heyden, Lipinski & Matthee, 2010) and other snappers from Northeastern Brazil and sympatric to *L. alexandrei*, like *L. synagris* (Silva et al., 2018). Consequently, these data are not suitable to diagnosis of pressure by fisheries since this is a recent activity as mentioned earlier (Evangelista-Gomes et al., 2020).

### Genetic connectivity

The present results indicated that *L. alexandrei* is composed of a single population, a trait putatively associated with the species-specific biological features as proposed for other snappers from Northeastern coast of Brazil, such as *L. purpureus*. In the latter, the genetic homogeneity was hypothetically explained by the presence of pelagic eggs and long period of larval development, as also reported in other Lutjanidae species from Atlantic Ocean (da Silva et al., 2016). Likewise, *O. chrysurus*, a closely related species to *Lutjanus*, distributed in sympatry with several species of the genus, is also composed of a panmictic population along the Brazilian coast, what has been associated with ecological traits such as the duration of larval stages (da Silva et al., 2015).

The type of eggs and larval periods, favored by the synergetic action of current tides, are features that could account for the panmixia in both *L. purpureus* and *O. chrysurus* from the Brazilian coast (da Silva et al., 2015, 2016). Most likely, the currents along the Brazilian coast could also potentialize the dispersal of larvae of *L. alexandrei*, a scenario particularly favored by the apparent lack of barriers to population subdivision of these snappers, as also suggested for other marine fishes (Palumbi, 1994).

Besides the features related to type of eggs and larvae and the role of Brazilian currents, the genetic connectivity found in *L. alexandrei* might be also influenced by the close relationship of this species with estuaries. Juveniles of Brazilian snapper usually inhabit coastal areas such as mangroves while adults are frequently observed in deeper reef environments (Aschenbrenner & Ferreira, 2015). This migration to both types of habitats might increase the admixture pattern reported in *L. alexandrei*, as also inferred for *L. jocu* from Northeastern Brazil and sympatric to *L. alexandrei* (Martins, 2018).

The hypotheses about the causes of the genetic connectivity within *L. alexandrei* could be more precise if other studies about the life traits of this species were available inasmuch as reliable phylogeographic inferences invariably rely on biological and ecological aspects of the analyzed organisms (Bowen et al., 2014). Unfortunately, there is no information whether the duration of the larval stages in *L. alexandrei* is extended or not, as previously reported in other sympatric snapper species like *L. analis, L. synagris, L. jocu* and *Rhomboplites aurorubens* (Lessa, Nóbrega & Bezerra, 2004; Freitas et al., 2011), or else if this species presents great dispersal abilities that could explain the high admixture among haplotypes.

### Demographic history

The negative values obtained for most populations by the neutrality tests based on the fragments used to compare the samples of *L. alexandrei* indicate deviations from neutrality, what could be related to population expansion events. This suggestion was corroborated by the results from EBSP inasmuch as a 10-fold increase in Ne (effective population size) of *L. alexandrei* has taken place about 800 thousand years ago.

According to Rabassa, Coronato & Salemme (2005) and Rabassa, Coronato & Martínez (2011), this period corresponds to the Middle Pleistocene, culminating in a glaciation. These authors highlighted that climate changes in this period influence the development of ecosystems in South America.

A study carried out by Costa et al. (2019) about the demographic changes in Neotropical fishes from Pleistocene revealed that the sea level along the Brazilian coast during the last glacial maximum was 120 m below the present level, thus exposing large areas of the continental shelf and putatively increasing the availability of brackish and freshwater habitats. Therefore, freshwater and estuarine species would benefit from new colonization sites and increased resources, thus leading to increased effective population sizes.

Partially similar results in relation to the present data were reported in other snappers that co-occur with *L. alexandrei*. Events of population expansion in *L. jocu* and *L. analis* were estimated between 36 to 55 thousand years ago and 13 to 61 thousand years ago, respectively (Souza et al., 2019). In the yellowtail snapper (*O. chrysurus*) from the Brazilian coast, a sudden increase of population size was also inferred during Pleistocene (da Silva et al., 2015), similarly to the pattern reported for *L. purpureus* (Gomes, Sampaio & Schneider, 2012; da Silva et al., 2016) and *L. synagris* (Silva et al., 2018).

Although other snappers distributed sympatrically with *L. alexandrei* also experienced population growth during the Pleistocene, these showed a much more recent population expansion than *L. alexandrei*. This is probably due to the fact that most studies reporting this increase in the effective number were based only on mitochondrial DNA (Souza et al., 2019; Gomes, Sampaio & Schneider, 2012) or used a mutation rate of 10% per million years (da Silva et al., 2015, 2016; Silva et al., 2018).

EBSP has its limitations, mainly in relation to the use of mutation rates between different molecular fragments, which can sometimes make it difficult to calibrate (Heled & Drummond, 2008), however this method has been widely used to estimate the demographic history of fish, including snappers (da Silva et al., 2015, 2016; Silva et al., 2018).

## Conclusions

This is the first study about the population structure and demographic history of *L. alexandrei*, a recently described species from Western South Atlantic and endemic to the Northeastern Brazilian coast. The results from both mitochondrial and nuclear fragments revealed a consistent pattern of genetic homogeneity for the populations of Brazilian snapper, suggesting *L. alexandrei* can be managed as a single stock across their range. This scenario agrees with that previously reported in other Lutjanidae species from the Brazilian coast, being associated to high levels of gene flow driven by larval dispersal and the role of sea currents along this region. However, basic information about the life traits of *L. alexandrei* that could provide insights to elucidate our findings are still missing.

High levels of genetic diversity were recovered in the Brazilian snapper, particularly based on analyses of CR, being even higher than those reported in Lutjanidae species from the northeastern coast of Brazil and sympatric to *L. alexandrei*. These results may be an alert regarding the diversity found for other species, such as *L. purpureus*, a highly commercialized species with evidence of overexploitation, since the apparent high levels of DNA polymorphism reported in these fishes might actually have been decreasing over time.

Finally, the inferences about the demographic history in Brazilian snapper revealed population expansion, possibly favored by the emergence of new habitats from sea level variation reported during Pleistocene.

## Additional Information and Declarations

### Field Study Permissions

The following information was supplied relating to field study approvals (*i.e*., approving body and any reference numbers): All samples were collected/transported with authorization of the Instituto Chico Mendes de Conservação da Biodiversidade (ICMBio) (SISBIO license n. 62250-1).

### Data Availability

The following information was supplied regarding data availability: The Cytochrome b sequences are available at GenBank: MZ343890–MZ343999. The Control Region sequences are available at GenBank: MZ327145–MZ327255. The NADH-dehydrogenase subunit 4 sequences are available at GenBank: MZ356036–MZ356152. The Intron 1 of the S7 ribosomal protein sequences are available at GenBank: MZ355926–MZ356035. The Intron 1 of the Myostatin sequences are available at GenBank: MZ344000–MZ344114.
